# MicroRNA-500 sustains nuclear factor-κB activation and induces gastric cancer cell proliferation and resistance to apoptosis

**DOI:** 10.18632/oncotarget.2800

**Published:** 2015-01-30

**Authors:** Liang Zhang, Ya Ding, Zhongyu Yuan, Junling Liu, Jian Sun, Fangyong Lei, Shu Wu, Su Li, Dongsheng Zhang

**Affiliations:** ^1^ State Key Laboratory of Oncology in Southern China, Collaborative Innovation Center for Cancer medcine, Department of Diagnostic Imaging and Interventional radiology, Sun Yat-sen University Cancer Center, Guangzhou 510060, China; ^2^ Department of Biotherapy, Sun Yat-sen University Cancer Center, Guangzhou 510060, China; ^3^ Department of Medical Oncology, Sun Yat-sen University Cancer Center, Guangzhou 510060, China; ^4^ Clinical Trial Center, Sun Yat-sen University Cancer Center, Guangzhou 510060, China; ^5^ Department of Experimental Research, Sun Yat-sen University Cancer Center, Guangzhou 510060, China

**Keywords:** miR-500, Gastric cancer, NF-κB signalling pathway, deubiquitinase

## Abstract

Ubiquitin deconjugation of key signalling molecules by deubiquitinases (DUBs) such as cylindromatosis (CYLD), A20, and OTU deubiquitinase 7B (OTUD7B) has emerged as an important regulatory mechanism in the downregulation of NF-κB signalling and homeostasis. However, how these serial negative regulations are simultaneously disrupted to result in constitutive activation of NF-κB signalling in cancers remains puzzling. Here, we report that the miR-500 directly repressed the expression of CYLD, OTUD7B, and the A20 complex component Tax1-binding protein 1 (TAX1BP1), leading to ubiquitin conjugation of receptor-interacting protein 1 (RIP1) and sustained NF-ĸB activation. Furthermore, we found that miR-500 promoted gastric cancer cell proliferation, survival, and tumorigenicity. Importantly, miR-500 was upregulated in gastric cancer and was highly correlated with malignant progression and poor survival. Hence, we report the uncovering of a novel mechanism for constitutive NF-κB activation, indicating the potentially pivotal role of miR-500 in the progression of gastric cancer.

## INTRODUCTION

Gastric cancer, one of the most aggressive malignancies of the gastrointestinal tract, is the fourth most common cancer and the second leading cause of cancer death worldwide [1, 2]. Despite advances in treatment modalities, the prognosis for gastric cancer patients has not been significantly improved, and the overall 5-year survival rate remains as poor as 10% to 40% [3]. Numerous studies have revealed that constitutively activated NF-ĸB signalling plays vital roles in the development and progression of gastric cancer, and blockade of the NF-κB pathway inhibits gastric cancer cell proliferation, sensitises cells to chemotherapeutic drugs, and even suppresses distant metastasis [4–7]. Hence, better understanding of the molecular mechanisms underlying NF-κB activation in gastric cancer may allow the identification of novel therapeutic targets for gastric cancer.

Over the last decade, it was found that ubiquitination occurs at multiple steps within the NF-ĸB signalling cascades, and has emerged as an important regulatory mechanism for NF-κB signalling [8, 9]. For instance, TNF-α binding to TNF receptor (TNFR) triggers the recruitment of TNFRSF1A-associated via death domain (TRADD), TNFR-associated factor 2 (TRAF2), cellular inhibitor of apoptosis 1 (cIAP1), cIAP2, and receptor-interacting protein 1 (RIP1) to form a receptor-associated complex [10]. RIP1 then rapidly undergoes K63-linked polyubiquitination at lysine 377 (K377) by TRAF2 or the cIAPs [11, 12]. The K63-linked polyubiquitin chains of RIP1 then serve as scaffolds facilitating the recruitment and activation of TGF-β–activated kinase 1 (TAK1) and IKK complexes by binding to TAK1-binding proteins (TABs) and NF-κB essential modulator (NEMO) [13]. Consequently, the activated IKK complex phosphorylates cytoplasmic IĸBs, leading to K48-linked polyubiquitination-mediated IκBs degradation and release of NF-κB to the nucleus, where it activates transcription of its target genes [14, 15]. Therefore, RIP1 is a key target of deubiquitinases (DUBs) that downregulate TNF-α–induced NF-κB signalling.

It has been determined that many DUBs, such as cylindromatosis (turban tumour syndrome) (CYLD), A20, and OTU deubiquitinase 7B (OTUD7B), negatively regulate NF-ĸB signalling by ubiquitin deconjugation of key signalling molecules such as RIP1 [8]. CYLD, a K63-specific DUB, inhibits NF-κB signalling transmission by specifically removing K63-linked polyubiquitin chains from multiple key intermediaries, including RIP1, NEMO, and B-cell CLL/lymphoma 3 (BCL3) [16–18]. A20 removal of K63-linked polyubiquitin chains of RIP1 relies on ubiquitin-binding adaptor proteins such as Tax1-binding protein 1 (TAX1BP1), and promotes RIP1 degradation by catalysing the formation of K48-linked polyubiquitin chains onto RIP1, leading to inhibition of NF-κB [19–21]. OTUD7B, a newly identified member of the A20 family of DUBs, inhibits RIP1 ubiquitination and downstream IKK activity and NF-κB signalling [22]. Consistent with their roles in NF-κB signalling inhibition, DUB downregulation is involved in the initiation and progression of human cancers [23–25]. However, how these serial negative regulators are simultaneously repressed to induce constitutive activation of NF-κB signalling in cancers remains puzzling.

It is widely acknowledged that miRNAs can potentially affect multiple steps of cancer progression and tumorigenesis by simultaneously repressing a variety of target genes by binding to their mRNA 3′ untranslated regions (3′ UTRs) [26, 27]. By analysing miRNA array data from The Cancer Genome Atlas (TCGA) and using real-time PCR, we found that miR-500 was significantly upregulated in human primary gastric cancer and was correlated with poor prognosis. We demonstrate that miR-500 directly represses multiple DUBs, leading to constitutive activation of NF-κB signalling and promoting gastric cancer malignant progression both *in vitro* and *in vivo*. Taken together, our results describe a novel regulatory mechanism for activating NF-κB signalling, suggesting that miR-500 is a functional oncogenic miRNA in gastric cancer progression.

## RESULTS

### Overexpression of miR-500 correlated with gastric cancer progression

**Figure 1:**
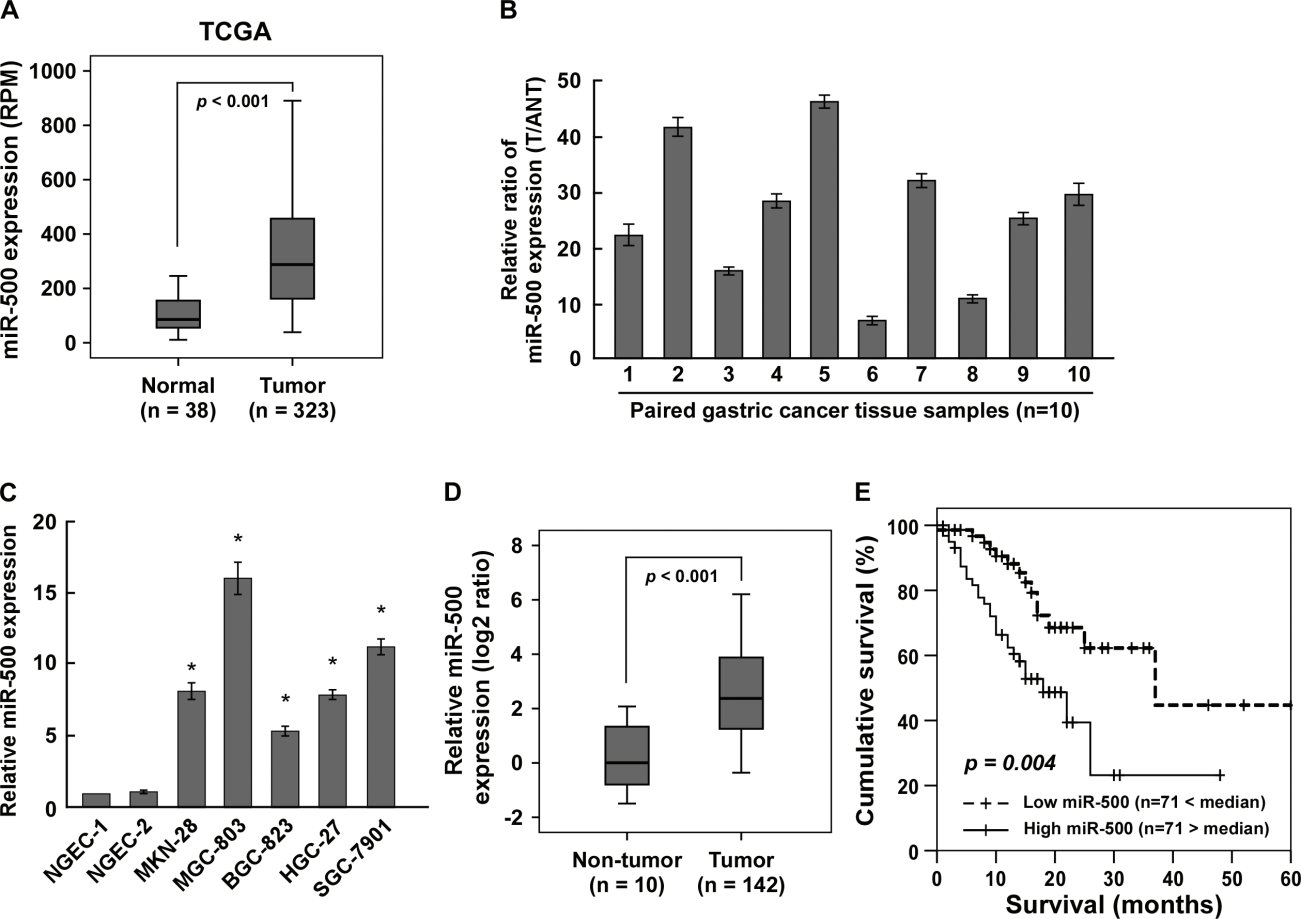
Overexpression of miR-500 correlates with gastric cancer progression **(A)** Analysis of TCGA miRNA array data revealing that miR-500 levels were significantly upregulated in human primary gastric cancer tissues (*n* = 323) compared with that in normal gastric tissues (*n* = 38) (*p* < 0.001). Real-time PCR analysis of miR-500 expression in **(B)** 10 pairs of gastric cancer samples (T) and adjacent normal tissues (ANT) and in **(C)** two NGEC lines and five cultured gastric cancer cell lines. Transcript levels were normalised by *U6* expression. **(D)** MiR-500 expression in 142 gastric cancer samples and 10 normal gastric tissues assessed by real-time PCR. Transcript levels were normalised by *U6* expression. Boundaries of boxes represent lower and upper quartiles, respectively. Lines within boxes and whiskers denote median and extremum, respectively. **(E)** Kaplan–Meier analysis of 5-year overall survival curves of gastric cancer patients with low miR-500 expression (<median, *n* = 71) or high miR-500 expression (>median, *n* = 71). Each bar represents the mean ± SD of three independent experiments. *p < 0.05.

Analysis of TCGA miRNA array data showed that miR-500 levels were significantly upregulated in human primary gastric cancer tissues (*n* = 323) compared with that in normal gastric tissues (*n* = 38) (*p* < 0.001) (Figure 1A). We verified this result with real-time PCR, finding that miR-500 levels were increased in the 10 gastric cancer tissues compared to the matched adjacent non-tumour tissues and in the five gastric cancer cell lines compared to the two normal gastric epithelial cells (NGEC-1 and NGEC-2) (Figure 1B and 1C). Collectively, these results indicate that miR-500 is upregulated in human gastric cancer.

We further evaluated whether miR-500 upregulation was clinically correlated with gastric cancer progression in the archived gastric cancer specimens. Figure 1D shows that miR-500 was markedly upregulated in gastric cancer samples compared to the 10 normal gastric samples. Statistical analysis revealed that miR-500 expression strongly correlated with clinical stage (*p* < 0.001), TNM classification (T: *p* < 0.001; N: *p* = 0.018; M: *p* = 0.001), and histological differentiation (*p* = 0.028) in the gastric cancer samples (Supplementary Table 1 and 2). Importantly, high miR-500 expression was associated with shorter overall survival in patients with primary gastric cancer (*p* < 0.001; Figure 1E), and miR-500 expression was identified as an independent prognostic factor (hazard ratio = 2.234, 95% CI = 1.662–3.232, *p* < 0.001; Supplementary Table 3). Taken together, these results suggest that miR-500 overexpression might be involved in human gastric cancer progression.

### Inhibition of miR-500 inhibited cell proliferation and induced apoptosis of gastric cancer cells *in vitro*

**Figure 2:**
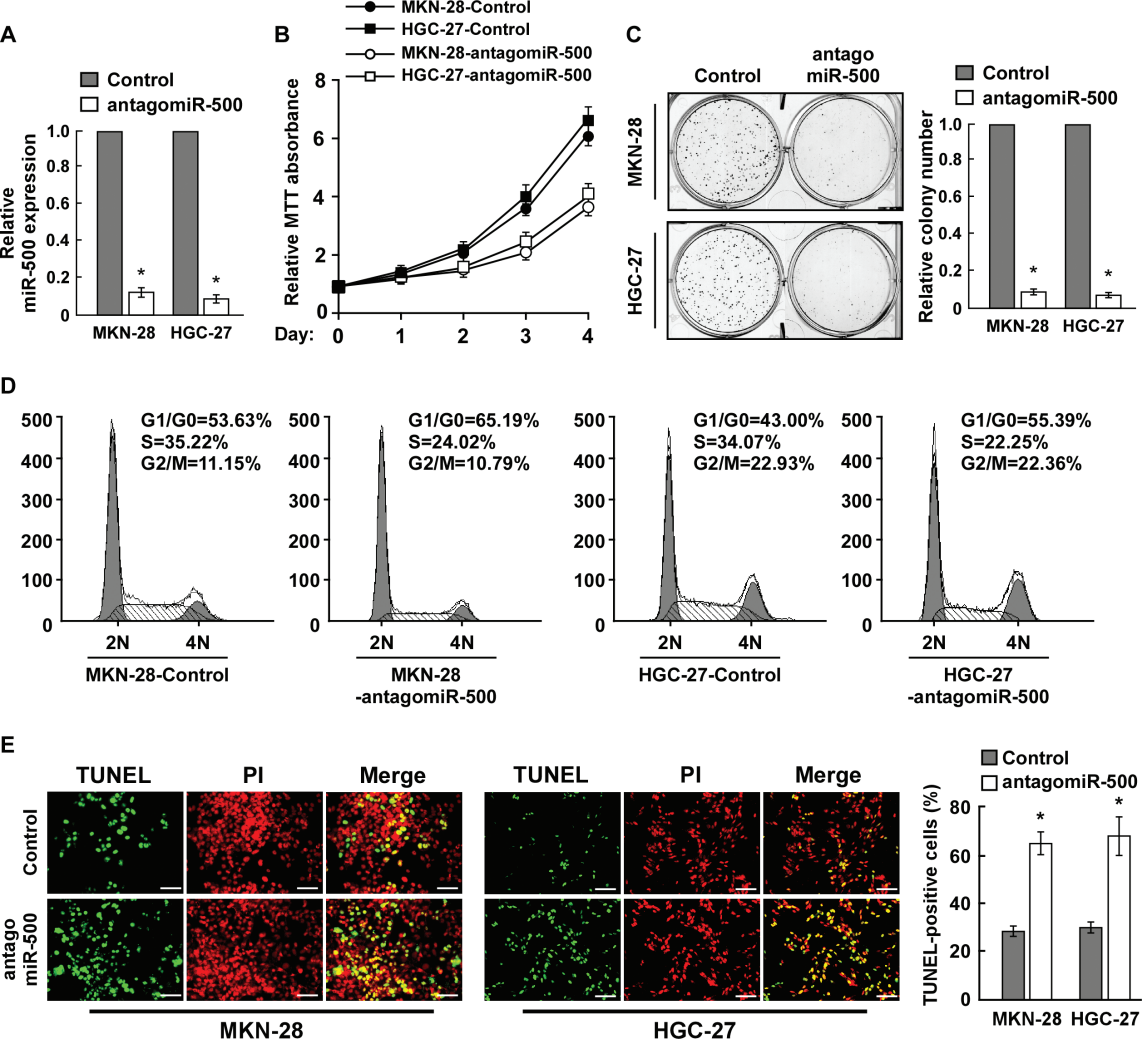
Inhibition of miR-500 suppresses proliferation and induces apoptosis of gastric cancer cells *in vitro* **(A)** Real-time PCR analysis of miR-500 expression in miR-500–silenced cells. Transcript levels were normalised by *U6* expression. **(B)** MTT assay revealing that miR-500 downregulation inhibited proliferation of MKN-28 and HGC-27 cells. **(C)** Representative micrographs (left) and quantification (right) of crystal violet–stained colonies. **(D)** Flow cytometry cell cycle analysis of gastric cancer cells. **(E)** Representative micrographs (left) and quantification of TUNEL-positive cells in cells treated with cisplatin (20 μM) for 36 h. Each bar represents the mean of three independent experiments. *p < 0.05.

To investigate the biological effect of miR-500 overexpression on gastric cancer progression, miR-500 expression was silenced by transfection of antagomiR-500 in MKN-28 and HGC-27 cells (Figure 2A). MTT and colony formation assays showed that miR-500 inhibition significantly reduced the proliferation rate of the MKN-28 and HGC-27 cells (Figure 2B and 2C). Moreover, flow cytometry showed that the percentage of cells in S phase was dramatically decreased in miR-500–silenced MKN-28 and HGC-27 cells compared with the controls (Figure 2D). Meanwhile, miR-500 silencing rendered gastric cancer cells more sensitive to treatment by the chemotherapeutic agent cisplatin, as indicated by the TUNEL and annexin V assays (Figure 2E and Supplementary Figure 1).

We established MKN-28 and HGC-27 cell lines that stably expressed miR-500 (Figure 2A). MTT and colony formation assays showed that miR-500 upregulation significantly increased the proliferation rate of the MKN-28 and HGC-27 cells (Figure 2B and 2C). Moreover, flow cytometry showed that the percentage of cells in S phase was dramatically increased in miR-500–overexpressing MKN-28 (50.30%) and HGC-27 (51.01%) cells compared with the controls (MKN-28 cells, 34.23%; HGC-27 cells, 33.22%; Figure 2D). Meanwhile, ectopically expressing miR-500 rendered gastric cancer cells more resistant to treatment by the chemotherapeutic agent cisplatin, as indicated by the TUNEL and annexin V assays (Figure 2E and Supplementary Figure 1).

Furthermore, we established MKN-28 and HGC-27 cell lines that stably expressed miR-500 (Supplementary Figure 2A). As expected, miR-500–overexpressing cells displayed increased proliferation rates and S phase cell cycle transition (Supplementary Figure 2B–2D). Ectopic expression of miR-500 rendered gastric cancer cells more resistant to treatment by the chemotherapeutic agent cisplatin, as indicated by the TUNEL and annexin V assays (Supplementary Figure 2E and 2F). Meanwhile, the effects of miR-500 on promoting cell proliferation and suppressing apoptosis were also observed in hepatocellular carcinoma cell line HepG2 (Supplementary Figure 3A–3C). Taken together, these results suggest that miR-500 promotes proliferation and reduces apoptosis of gastric cancer cells *in vitro*.

### MiR-500 overexpression contributed to gastric cancer progression *in vivo*

**Figure 3:**
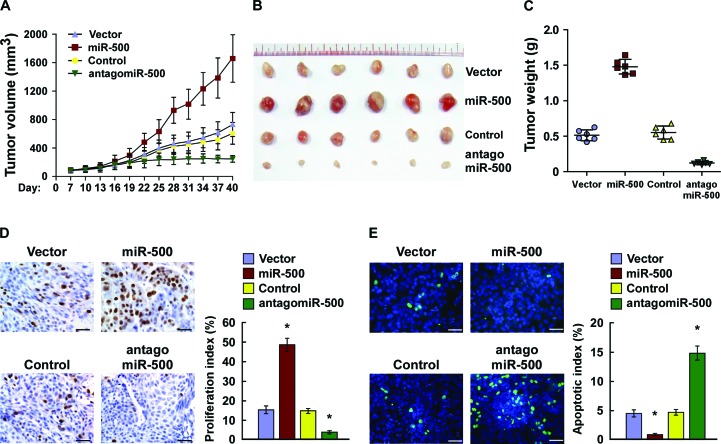
Overexpression of miR-500 contributes to gastric cancer progression *in vivo* **(A)** Xenograft model in nude mice. Tumour volumes were measured on the indicated days and presented as the mean ± SD. **(B)** Images of tumours from all mice in each group. **(C)** Tumour weights of each group. **(D)** Proliferation index determined by counting the proportion of Ki67-positive cells. **(E)** Apoptotic index measured by the percentage of TUNEL-positive cells. Scale bars: 50 μm. Each bar represents the mean ± SD of three independent experiments. *p < 0.05.

We examined the oncogenic role of miR-500 in gastric cancer progression using an *in vivo* tumour model. As shown in Figure 3A–3C, miR-500–overexpressing tumours were significantly larger, in both size and weight, than control tumours. Importantly, intratumoral injection of antagomiR-500 dramatically inhibited tumour growth, but injection of the antagomiR control had no effect on tumour development (Figure 3A–3C). Consistently, the miR-500 expression was significantly increased in the miR-500–overexpressing tumours but decreased in miR-500–silenced tumours (Supplementary Figure 4A). Furthermore, western blotting analysis revealed that the expression of CYLD, TAX1BP1 and OTUD7B dramatically decreased in the miR-500–overexpressing tumours but increased in miR-500–silenced tumours (Supplementary Figure 4B). Meanwhile, the staining assays revealed that miR-500–overexpressing tumours had increased percentages of Ki67-positive cells and decreased percentages of TUNEL-positive cells, whereas miR-500–silenced tumours had a lower Ki67 proliferation index and a higher percentage of TUNEL-positive apoptotic cells (Figure 3D and 3E). Therefore, our results suggest that miR-500 overexpression contributes to gastric cancer progression *in vivo*.

### MiR-500 activated the NF-ĸB signalling pathway

**Figure 4:**
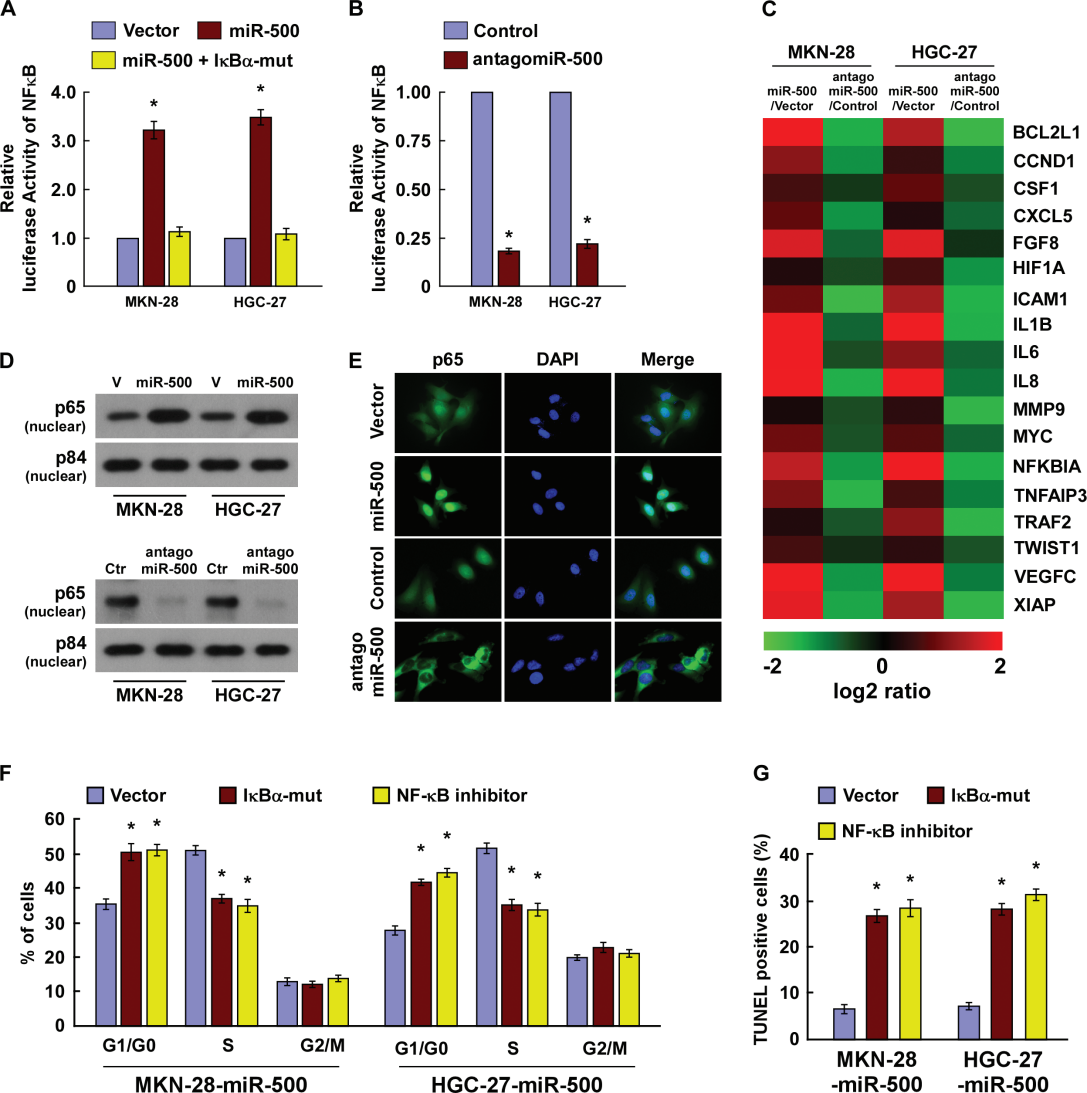
MiR-500 activates the NF-ĸB signalling pathway **(A)** NF-κB luciferase reporter activities were analysed in miR-500 or miR-500 plus IκBα-mut cells. **(B)** NF-κB luciferase reporter activities were analysed in miR-500–inhibited cells. **(C)** Real-time PCR analysis indicating an apparent overlap between NF-κB–dependent gene expression and miR-500–regulated gene expression. The pseudocolour represents the intensity scale of miR-500 versus Vector (V) or antagomiR-500 versus Control (Ctr), generated by log2 transformation. **(D)** Western blotting of nuclear p65 expression. The nuclear protein p84 was used as a nuclear protein marker. **(E)** Immunofluorescence staining of subcellular localisation of NF-κB/p65. **(F)** Cell cycle analysis of MKN-28 cells transfected with miR-500 or miR-500 plus IκBα-mut, or miR-500 plus NF-κB inhibitor (JSH-23). **(G)** TUNEL of MKN-28 cells transfected with miR-500 or miR-500 plus IκBα-mut, or miR-500 plus NF-κB inhibitor (JSH-23), and treated with cisplatin (20 μM) for 36 h. Each bar represents the mean ± SD of three independent experiments. *p < 0.05.

We next explored the underlying molecular mechanism that might be responsible for the oncogenic roles of miR-500. Since NF-ĸB signaling pathway is one of the key signalling pathways that contributes to cell proliferation and apoptosis [29], and has been found frequently hyperactivated in gastric cancers [4], we then examined whether miR-500 regulated the NF-κB activity. As expected, overexpression of miR-500 significantly increased, but silencing of miR-500 reduced, the NF-ĸB luciferase activity and the expression levels of numerous NF-ĸB downstream target genes in gastric cancer cells (Figure 4A–4C). And transfection of a IκBα dominant-negative mutant (IκBα-mut) abrogated NF-ĸB activation induced by miR-500 overexpression (Figure 4A). Moreover, cellular fractionation and immunofluorescence staining assays showed that miR-500 overexpression promoted nuclear accumulation of NF-κB/p65, while miR-500 silencing reduced nuclear NF-κB/p65 expression but not total p65 level (Figure 4D, 4E and Supplementary Figure 5), indicating that miR-500 activates the NF-κB signalling pathway by promoting nuclear NF-κB/p65 accumulation. Importantly, the stimulatory effects of miR-500 on gastric cancer cell proliferation and survival were markedly reduced upon NF-κB inhibition by transfection of IκBα-mut or treatment with a NF-κB inhibitor (Figure 4F and 4G). Thus, these results demonstrate that functional activation of the NF-κB signalling pathway is vital to the biological effects of miR-500 in gastric cancer progression.

### MiR-500 directly suppressed multiple NF-ĸB negative regulatory genes

**Figure 5:**
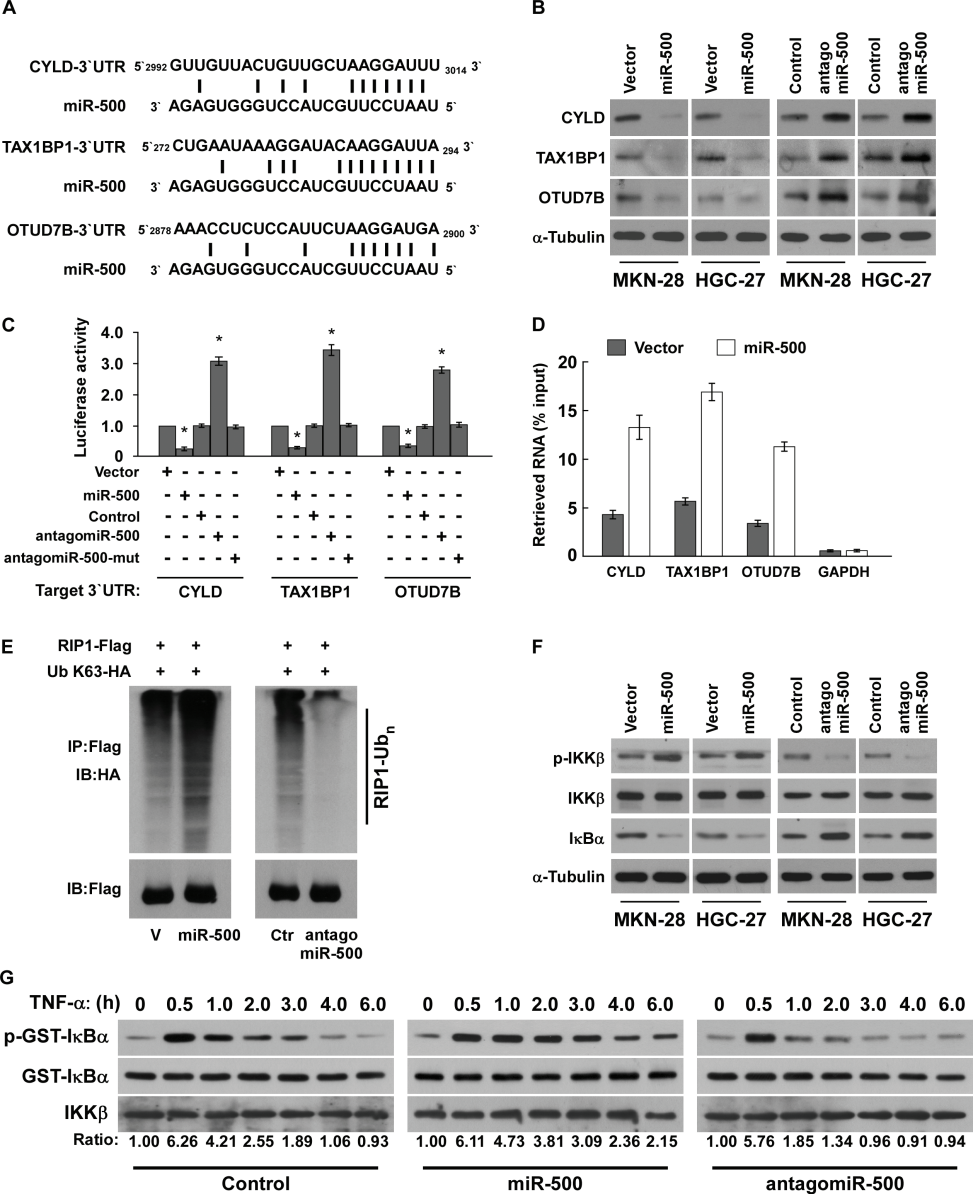
MiR-500 directly suppresses multiple NF-ĸB negative regulatory genes **(A)** Predicted miR-500 target sequence in 3′ UTRs of CYLD, TAX1BP1, and OTUD7B. **(B)** Western blots of CYLD, TAX1BP1, and OTUD7B expression. α-Tubulin served as the loading control. **(C)** Luciferase assay of cells transfected with pGL3-CYLD-3′UTR, pGL3-TAX1BP1-3′UTR, or pGL3-OTUD7B-3′UTR reporter with miR-500 mimic, antagomiR-500, or miR-500-mut mimic. **(D)** MiRNP IP assay showing the association between miR-500 and CYLD, TAX1BP1, and OTUD7B transcripts. *GAPDH* served as the negative control. **(E)** Western blots of K63-linked polyubiquitin levels of RIP1 in miR-500–overexpressing or miR-500–silenced MKN-28 cells treated with TNF-α (10 ng/mL). V, vector; Ctr, control. **(F)** Western blot analysis of p-IKKβ, total IKKβ, and IκBα expression in cells treated with 10 ng/ml TNF-α. **(G)** *In vitro* IKK kinase assay of vector- or miR-500–overexpressing cells, or miR-500–silenced cells treated with 10 ng/mL TNF-α. IKKβ was subjected to IP, and kinase activity was determined by phosphorylation of a recombinant GST-IκBα substrate using a phospho-specific IκαB antibody. The equal IP of IKKβ was shown. Each bar represents the mean ± SD of three independent experiments. *p < 0.05.

Using the TargetScan program, we found that CYLD, OTUD7B, and the A20 complex component TAX1BP1, which function as critical negative regulatory genes by deconjugating K63-polyubiquitin chains from RIP1 [21, 28], might be potential targets of miR-500 (Figure 5A). Western blot analysis revealed that CYLD, TAX1BP1, and OTUD7B expression levels were significantly decreased in miR-500–transduced cells, but were increased in miR-500–silenced cells (Figure 5B), suggesting that miR-500 negatively regulated these proteins. Furthermore, luciferase assay indicated that miR-500 overexpression decreased the reporter activities linked with the 3′ UTR of their transcripts, but miR-500 silencing increased it (Figure 5C). However, ectopically expressing the miR-500 mutant (miR-500-mut) did not result in repressive effects on the 3′ UTRs (Figure 5C). Importantly, microribonucleoprotein (miRNP) IP showed that miR-500 overexpression enriched the transcripts of CYLD, TAX1BP1, and OTUD7B, but not GAPDH, that assembled into the miRNP complexes, indicating that miR-500 directly targets the mRNA 3′ UTR regions of these transcripts (Figure 5D). Taken together, our results demonstrate that CYLD, TAX1BP1, and OTUD7B are *bona fide* targets of miR-500.

### MiR-500 promoted ubiquitin conjugation of RIP1 and sustained NF-ĸB activity

As the inhibitory effect of CYLD, TAX1BP1, and OTUD7B on NF-ĸB activation are associated with deconjugation of K63-polyubiquitin chains from RIP1 [19, 21, 22], we then examined the effect of miR-500 on the ubiquitination status of RIP1. As shown in Figure 5E, miR-500 overexpression drastically increased the K63-polyubiquitin levels of RIP1 in gastric cancer cells, but miR-500 inhibition decreased it. Concordantly, miR-500 overexpression led to elevated phosphorylation of IKKβ and reduced IκBα, which was abrogated by the antagomiR-500 (Figure 5F). Furthermore, *in vitro* IKK kinase assay revealed that activation of the IKK kinase complex induced by TNF-α treatment was prolonged in miR-500–overexpressing cells, but was rapidly decreased in miR-500–inhibited cells (Figure 5G), suggesting that miR-500 overexpression sustains NF-ĸB activation in gastric cancer cells. Importantly, the repressive effect of antagomiR-500 on NF-κB activity and BCL2L1, CCND1 expression were potently antagonised by individual silencing of CYLD, TAX1BP1, or OTUD7B (Supplementary Figure 6A and 6B), indicating that CYLD, TAX1BP1, and OTUD7B are functionally relevant effectors of miR-500 in NF-κB activation.

### Clinical relevance of miR-500, NF-κB activation, and its targets in gastric cancer

**Figure 6:**
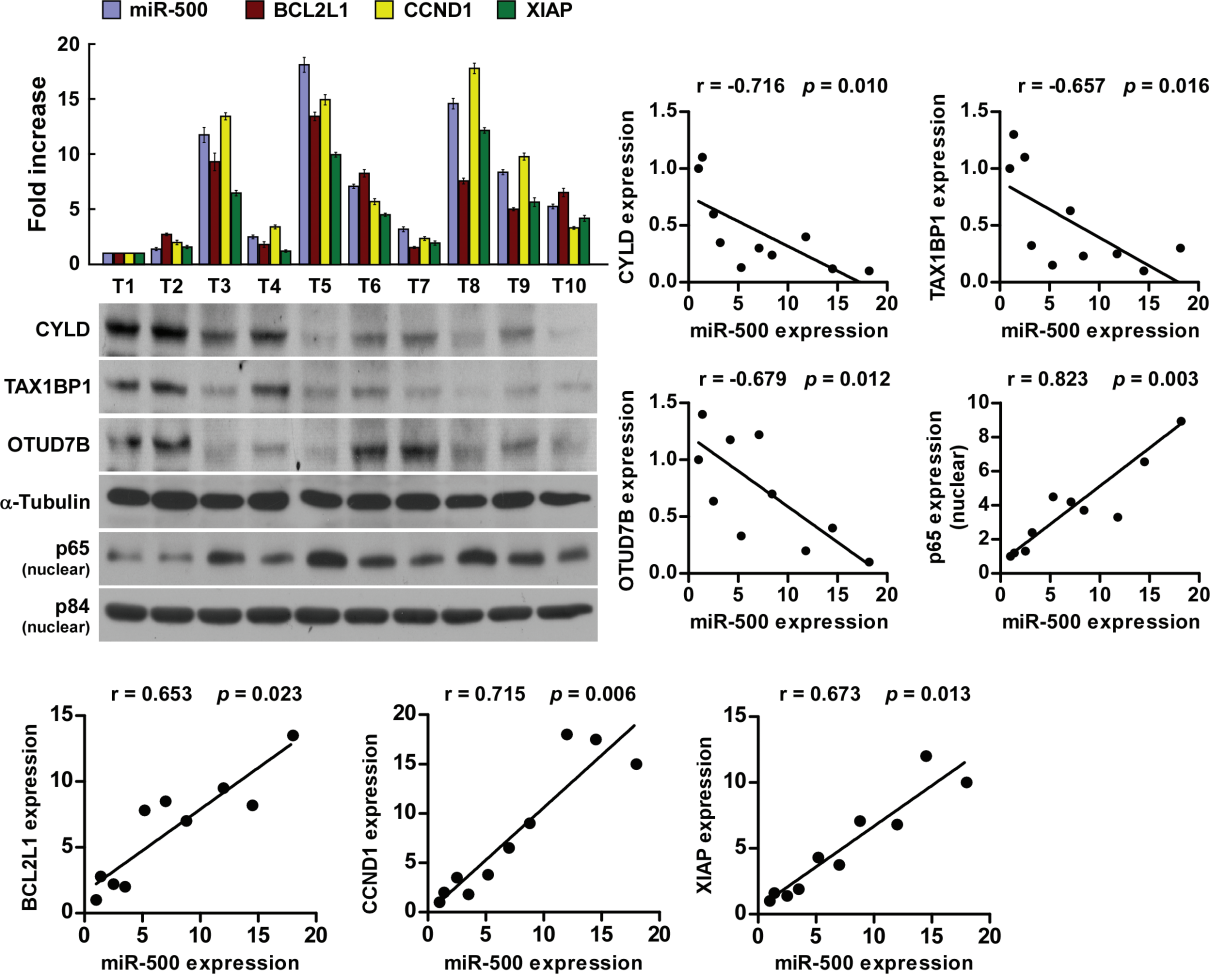
Clinical relevance of miR-500, NF-κB activation, and its targets in gastric cancer Analysis (left) and correlation (right) of miR-500 expression and CYLD, TAX1BP1, OTUD7B, nuclear p65 expression, and *BCL2L1*, *CCND1*, and *XIAP* mRNA levels in 10 freshly collected human gastric cancer tissue samples (T). α-Tubulin and nuclear protein p84 were used as loading controls. The ratio of first sample (CYLD/α-tubulin, TAX1BP1/α-tubulin, OTUD7B/α-tubulin, nuclear p65/p84) was considered as 1.0. Each bar represents the mean ± SD of three independent experiments.

Lastly, we examined whether miR-500–mediated suppression of CYLD, TAX1BP1, and OTUD7B, and NF-κB activity in gastric cancers are clinically relevant. As shown in Figure 6, miR-500 levels in the 10 freshly collected gastric cancer samples were inversely correlated with the expression levels of CYLD (r = −0.716, *p* = 0.010), TAX1BP1 (r = −0.657, *p* = 0.016), and OTUD7B (r = −0.679, *p* = 0.012), but were positively correlated with nuclear p65 expression (r = 0.823, *p* = 0.003) and mRNA levels of the NF-κB downstream genes BCL2-like 1 (*BCL2L1*) (r = 0.653, *p* = 0.023), cyclin D1 (*CCND1*) (r = 0.715, *p* = 0.006), and X-linked IAP (*XIAP*) (r = 0.673, *p* = 0.013). Taken together, our results suggest that miR-500 overexpression activates the NF-κB signalling pathway by repressing CYLD, TAX1BP1, and OTUD7B, and consequently results in gastric cancer aggressiveness and poor clinical outcomes.

## DISCUSSION

Constitutive activation of NF-κB signalling occurs commonly in human cancers and plays a critical role in cancer development and progression [29, 30]. NF-κB signalling transmission requires the ubiquitination of key signalling molecules by E3 ubiquitin ligases, while NF-κB activation is largely restricted by ubiquitin deconjugation induced by DUBs such as CYLD, A20, and OTUD7B [8]. However, how cancer cells override this negative regulation and induce sustained NF-κB activation remains largely unknown. Herein, we demonstrate that substantially overexpressed miR-500 activates and sustains NF-κB signalling by directly suppressing multiple NF-κB negative regulators, including CYLD, OTUD7B, and the A20 complex component TAX1BP1. Thus, our findings describe a novel mechanism that disrupts DUB negative regulation simultaneously, leading to constitutive NF-κB activation in cancer cells. Such a miRNA-mediated mechanism would be favourable for enhancing the oncogenic NF-κB signalling activation induced by extrinsic stimuli such as inflammatory cytokines to promote cancer progression.

It has been demonstrated that DUB malfunction plays a central role in tumorigenesis by promoting cell proliferation and survival, and even distant metastasis via activation of NF-κB signalling. CYLD was originally characterised as a tumour suppressor that is mutated in hereditary cylindromatosis, and was later found to be downregulated in many other cancers via deletion or transcriptional repression [31–34]. Similarly, there is downregulation of OTUD7B and TAX1BP1 in various cancers, which greatly contributes to hyperactivation of NF-κB signalling [23–25]. Herein, analysis by publicly available algorithms and our serial experimental results demonstrate that CYLD, TAX1BP1, and OTUD7B are *bona fide* targets of miR-500 in gastric cancer. Therefore, our results suggest a novel mechanism of DUB dysregulation involving CYLD, A20, and OTUD7B in gastric cancer and provide a functionally and clinically relevant epigenetic mechanism in cancer progression.

Despite therapeutic advances, the prognosis of gastric cancer remains poor largely due to the insensitivity to chemotherapeutic drugs and frequent relapse [35]. Given its critical role in cell survival, the NF-κB signalling pathway is considered a therapeutic target for gastric cancer. Thus, further understanding of this novel regulatory mechanism of NF-κB signalling may provide new clues for therapeutic intervention. We found that miR-500 overexpression activated NF-κB activity, while miR-500 inhibition markedly repressed it. MiR-500 silencing reduced *BCL2L1* and *XIAP* expression. Moreover, antagonising miR-500 rendered gastric cancer cells more sensitive to cisplatin treatment *in vitro* and promoted apoptosis *in vivo*. Thus, our results suggest the important role of miR-500 in gastric cancer chemoresistance via activation of NF-κB signalling.

Indeed, NF-κB inhibitors such as tipifarnib (CPT-11) and bortezomib (PS-341) have been developed and evaluated in phase I and phase II clinical trials [36, 37]. However, NF-κB signalling is also essential for normal cellular processes, including inflammation and immunity; therefore, molecules targeting NF-κB are likely to raise unfavourable pharmacokinetic property and toxicity concerns [38]. As the malfunction of DUBs such as CYLD, A20, and OTUD7B has been observed in various cancers and greatly contributes to constitutive NF-κB signalling activation, identifying the key factor(s) that upregulate and/or activate these DUBs might facilitate the identification of novel targets for therapeutic intervention. We found that silencing endogenous miR-500 upregulated the expression level or activity of multiple DUBs and reduced the strength and duration of NF-κB signalling. AntagomiR-500 inhibited the tumorigenicity of the gastric cancer cells. Importantly, we also found that miR-500 expression was markedly upregulated in gastric cancer tissues but remained comparatively low in normal gastric tissues. Therefore, our results suggest that miR-500 may represent a promising therapeutic target in cancer.

In summary, our study reveals that miR-500 overexpression plays important roles in gastric cancer progression and that miR-500 is a critical activator of NF-κB signalling. Understanding the precise role of miR-500 in the pathogenesis of gastric cancer and activation of the NF-κB signalling pathway will increase our knowledge of the biological basis of cancer progression and may also facilitate the development of new therapeutic strategies against gastric cancer.

## MATERIALS AND METHODS

### Cell culture

Two cultures of primary normal human gastric epithelial cells (NGEC-1 and NGEC-2) were established from gastric biopsy specimens obtained from upper gastrointestinal endoscopy and cultured as described previously [28]. The gastric cancer cell lines MKN-28, MGC-803, BGC-823, HGC-27, and SGC-7901 were obtained from American Type Culture Collection (Manassas, VA, USA) and maintained in DMEM (Invitrogen, Carlsbad, CA, USA) supplemented with 10% FBS (HyClone, Logan, UT, USA).

### Tissue specimens and clinicopathological characteristics

The 142 paraffin-embedded, archived gastric cancer samples used in this study were histopathologically and clinically diagnosed at the Sun Yat-sen University Cancer Center between 2008 and 2011. Clinical staging and clinicopathological tumour-nodes-metastasis (TNM) classification were determined according to the criteria proposed by the International Union Against Cancer. Written informed consent was obtained from all patients prior to the study. The Institutional Research Ethics Committee approved the use of the clinical specimens for research purposes. The clinicopathological characteristics of the samples are summarised in Supplementary Table 1. Freshly collected gastric cancer tissue specimens and matched adjacent non-tumour gastric tissue specimens were each collected from 10 patients, and were frozen and stored in liquid nitrogen until used. The correlation between miR-500 and the target genes was determined using another 10 freshly collected gastric cancer tissues. The information of these samples, including Clinical stage I: 2, Clinical stage II: 4, Clinical stage III: 2, has been added into material and method section in the revised manuscript.

### Plasmid, siRNA, and transfection

The human miR-500 gene was PCR-amplified from genomic DNA and cloned into a pMSCV-puro retroviral vector. pNF-κB-luc and control plasmids (Clontech) was used to quantitatively examine NF-κB activity. pBabe-Puro-IĸBα-mut (plasmid 15291) expressing IĸBα dominant-negative mutant (IĸBα-mut) was purchased from Addgene (Cambridge, MA).

We purchased miR-500 mimic, miR-500 antagonist (antagomiR-500), antagomiR-500-mut and controls from RiboBio (Guangzhou, China). Transfection of the plasmids, siRNAs, miR-500 mimic, and antagomiR-500 were performed using Lipofectamine 2000 (Invitrogen) according to the manufacturer's instructions. Stable cell lines expressing miR-500 were generated via retroviral infection using HEK293T cells and selected with 0.5μg/ml puromycin for 10 days.

The 3′UTR region of human CYLD, TAX1BP1 and OTUD7B, generated by PCR amplification from NHA, were cloned into the pGL3 luciferase reporter plasmid (Promega, Madison, WI). The cloning primers were as followed: CYLD-3′UTR-up: 5′-GCCCCGCGGTGTTCCTCACCTCCAAATAAA-3′; CYLD-3′UTR-dn: 5′-GCCCTGCAGAGGCACCTA GTA AGTGTCCG-3′; TAX1BP1-3′UTR-up: 5′-GCCCCGC GGCATAGAGCGGATGCTTTCA-3′; TAX1BP1-3′UTR-dn: 5′-GCCCTGCAGACCTATCATTTCATGGGCTA A-3′; OTUD7B-3′UTR-up: 5′-GCCCCGCGGTTCCGT TTGCTTTATTTTCA-3′; OTUD7B-3′UTR-dn: 5′-GCCC TGCAGCATGGGCTTGCCTCTTCTA-3′.

### Western blotting

Cells were harvested in cell lysis buffer (Cell Signaling Technology, Danvers, MA, USA) and heated for 5 min at 100°C. Equal quantities of denatured protein samples were resolved on 10% SDS-polyacrylamide gels, and then transferred onto PVDF membranes (Roche, Basel, Switzerland). After blocking with 5% non-fat dry milk in TBS/0.05% Tween 20, membranes were incubated with a specific primary antibody, followed by a horseradish peroxidase–conjugated secondary antibody. Proteins were visualised using ECL reagents (Pierce, Rockford, IL, USA). Antibodies against p65, p84, CYLD, TAX1BP1, and OTUD7B were purchased from Abcam (Cambridge, MA, USA). The membranes were stripped and reprobed with an anti– α-tubulin antibody (Sigma-Aldrich, St. Louis, MO, USA) as the loading control.

### MiRNA extraction and real-time quantitative PCR

Total miRNA from cultured cells and fresh surgical gastric tissues was extracted using a mirVana miRNA Isolation Kit (Ambion, Austin, TX, USA) according to the manufacturer's instructions. We synthesised cDNA from 10 ng total RNA using a TaqMan miRNA reverse transcription kit (Applied Biosystems, Foster City, CA, USA), and quantified the expression levels of miR-500 using a miRNA-specific TaqMan MiRNA Assay Kit (Applied Biosystems). The expression of miRNA was defined based on the Ct, and relative expression levels were calculated as 2^−[(Ct of miR-500) − (Ct of U6)]^ after normalisation with reference to expression of U6 small nuclear RNA.

### Cell cycle analysis

Cells were trypsinised and washed in ice-cold PBS, then fixed in ice-cold 80% ethanol in PBS. Bovine pancreatic RNase (Sigma-Aldrich) was added to a final concentration of 2 μg/mL, and cells were incubated at 37°C for 30 min, followed by incubation with 20 μg/mL propidium iodide (PI; Sigma-Aldrich) for 20 min at room temperature. Cell cycle profiles of 5 × 10^4^ cells were analysed using a FACSCalibur flow cytometer (BD Biosciences, San Jose, CA, USA).

### TUNEL

Apoptotic DNA fragmentation was examined using an *in situ* DeadEnd™ Fluorometric TUNEL System assay kit (Promega, Madison, WI, USA) according to the manufacturer's protocol. Briefly, cells were plated in 24-well flat-bottom plates and treated with cisplatin (10 μM) for 36 h. Cells were fixed in 4% paraformaldehyde at 4°C for 30 min, permeabilised in 0.1% Triton X-100, and labelled with fluorescein-12-dUTP using terminal deoxynucleotidyl transferase. The localised green fluorescence of apoptotic cells from the fluorescein-12-dUTP was detected by fluorescence microscopy (Axiovert 100M, Zeiss, Oberkochen, Germany).

### Xenografted tumour model and staining

BALB/c-nu mice (5–6 weeks old, 18–20 g) were purchased from the Experimental Animal Center of the Guangzhou University of Chinese Medicine and housed in barrier facilities on a 12-h light/dark cycle. The Institutional Animal Care and Use Committee of Sun Yat-sen University approved all experimental procedures. The mice were randomly assigned to groups (*n* = 6/group). The mice in two groups were inoculated subcutaneously with MKN-28/vector cells (5 × 10^6^) or with MKN-28/miR-500 cells (5 × 10^6^) in the left dorsal flanks, respectively. The mice in another two groups were inoculated subcutaneously with MKN-28 cells (5 × 10^6^) in the left dorsal flank, and Seven days later, injected intratumorally with 100 μL antagomiR control or antagomiR-500 (diluted in PBS at 2 mg/mL) three times per week for two weeks. Tumours were examined twice weekly; length, width, and thickness were measured with callipers, and tumour volumes were calculated. Tumour volume was calculated using the equation (L × W^2^)/2. On day 40, the animals were euthanised, and the tumours were excised, weighed, and paraffin-embedded. Serial 6.0-μm sections were cut and subjected to staining assays. The proliferation index was determined by counting the proportion of Ki67-positive cells. The apoptotic index was measured based on the percentage of TUNEL-positive cells.

### Luciferase assays

Cells (4 × 10^4^) were seeded in triplicate in 24-well plates and cultured for 24 h. Cells were transfected with 100 ng NF-κB reporter luciferase plasmid, or pGL3-CYLD-3′UTR, pGL3-TAX1BP1-3′UTR, or pGL3-OTUD7B-3′UTR luciferase plasmid, plus 5 ng pRL-TK *Renilla* plasmid (Promega) using Lipofectamine 2000 (Invitrogen) according to the manufacturer's recommendation. Luciferase and *Renilla* signals were measured 36 h after transfection using a Dual Luciferase Reporter Assay Kit (Promega) according to the manufacturer's protocol.

### Nuclear/cytoplasmic fractionation

Cells were washed with cold PBS and resuspended in buffer containing 10 mM HEPES (pH 7.8), 10 mM KCl, 0.1 mM EDTA, 1 mM Na_3_VO_4_, 1 mM DTT, 1:500 protease inhibitors (Sigma-Aldrich), and 0.2 mM PMSF, and incubated on ice for 15 min. Detergent was added and cells were vortexed for 10 s at the highest setting. Nuclei and the supernatant (“cytoplasm”) were separated by centrifugation at 4°C. Nuclei were resuspended in buffer containing 20 mM HEPES (pH 7.8), 0.4 M NaCl, 1 mM EDTA, 1 mM Na_3_VO_4_, 1 mM DTT, and 1:500 protease inhibitors and incubated on ice for 15 min. Nuclear extracts were collected by centrifugation at 14,000 × *g* for 10 min at 4°C.

### miRNP immunoprecipitation

miR-500-overexpressing and control cells were transfected with HA-Ago1, followed by HA-Ago1 immunoprecipitation using HA-antibody. Real-time PCR analysis of the IP material was used to test the association of the mRNA of CYLD, TAX1BP1, OTUD7B with the RISC complex. The GAPDH gene was used as a negative control.

### Ubiquitination assay

Cells were co-transfected with plasmids of RIP1-Flag, K63 specific ubqutin plasmid (Ub k63-HA). 24 h later, cells were treated with TNFα (10ng/ml). After stimulation, cells were rinsed with ice-cold PBS and lysed with the lysis buffer (25 mM HEPES [pH 7.4], 150 mM NaCl, 1% NP-40, 1 mM EDTA, 2% glycerol, 1 mM PMSF). The lysates were immunoprecipitated with the anti-Flag antibody (Santa Cruz Biotech., Santa Cruz, CA). and followed by the Flag immunoprecipitations. The immunoprecipitates were washed and followed by the western blot analysis using the anti-HA and anti-Flag antibodies (Sigma-Aldrich, St. Louis, MO, USA).

### IKK *in vitro* kinase assay

For immunoprecipitations, cells (5 × 10^6^) were either untreated or treated with TNFα (10ng/ml) in the absence of serum. After stimulation, cells were immediately rinsed with ice-cold PBS and lysed with the lysis buffer (25 mM HEPES [pH 7.4], 150 mM NaCl, 1% NP-40, 1 mM EDTA, 2% glycerol, 1 mM PMSF). The lysates were immunoprecipitated with the anti-IKKβ antibody (Santa Cruz Biotech., Santa Cruz, CA). The immunoprecipitates were washed with the lysis buffer extensively, followed by three washes with the kinase buffer, which contains 20 mM HEPES(pH 7.7), 2 mM MgCl_2_, 2 mM MnCl_2_, 100 mM NaCl, EDTA free proteinase inhibitor cocktail, 10 mM β-glycerophosphate, 10 mM Sodium orthovanadate, 10 mM p-nitrophenylphosphate, and 1 mM DTT. Recombinant GST-IĸBα (millipore, Billerica, MA) was used as the substrate for this assay incubated in kinase buffer with 500 μM ATP at 30°C for 30 min. Phosphorylation of the GST-IĸBα substrate was assayed by western blotting with p-IĸBα antibody(Cell Signaling, Danvers, MA).

### Statistical analysis

All statistical analyses were carried out using SPSS 13.0 statistical software (SPSS Inc., Chicago, IL, USA). Survival curves were plotted using the Kaplan–Meier method and compared by log-rank test. The 2-tailed Student's *t*-test was used to evaluate the significance of differences between two groups of data in all pertinent experiments. A *p*-value < 0.05 was considered significant.

## SUPPLEMENTARY MATERIALS AND METHODS FIGURES AND TABLES



## References

[1] Parkin DM, Bray F, Ferlay J, Pisani P (2005). Global cancer statistics, 2002. CA: a cancer journal for clinicians.

[2] Parkin DM (2004). International variation. Oncogene.

[3] Crew KD, Neugut AI (2006). Epidemiology of gastric cancer. World journal of gastroenterology: WJG.

[4] Sasaki N, Morisaki T, Hashizume K, Yao T, Tsuneyoshi M, Noshiro H, Nakamura K, Yamanaka T, Uchiyama A, Tanaka M, Katano M (2001). Nuclear factor-kappaB p65 (RelA) transcription factor is constitutively activated in human gastric carcinoma tissue. Clinical cancer research : an official journal of the American Association for Cancer Research.

[5] Sakamoto K, Hikiba Y, Nakagawa H, Hayakawa Y, Yanai A, Akanuma M, Ogura K, Hirata Y, Kaestner KH, Omata M, Maeda S (2010). Inhibitor of kappaB kinase beta regulates gastric carcinogenesis via interleukin-1alpha expression. Gastroenterology.

[6] Manu KA, Shanmugam MK, Ramachandran L, Li F, Fong CW, Kumar AP, Tan P, Sethi G (2012). First evidence that gamma-tocotrienol inhibits the growth of human gastric cancer and chemosensitizes it to capecitabine in a xenograft mouse model through the modulation of NF-kappaB pathway. Clinical cancer research : an official journal of the American Association for Cancer Research.

[7] Lin MT, Zuon CY, Chang CC, Chen ST, Chen CP, Lin BR, Wang MY, Jeng YM, Chang KJ, Lee PH, Chen WJ, Kuo ML (2005). Cyr61 induces gastric cancer cell motility/invasion via activation of the integrin/nuclear factor-kappaB/cyclooxygenase-2 signaling pathway. Clinical cancer research : an official journal of the American Association for Cancer Research.

[8] Harhaj EW, Dixit VM (2011). Deubiquitinases in the regulation of NF-kappaB signaling. Cell research.

[9] Liu S, Chen ZJ (2011). Expanding role of ubiquitination in NF-kappaB signaling. Cell research.

[10] Micheau O, Tschopp J (2003). Induction of TNF receptor I-mediated apoptosis via two sequential signaling complexes. Cell.

[11] Ea CK, Deng L, Xia ZP, Pineda G, Chen ZJ (2006). Activation of IKK by TNFalpha requires site-specific ubiquitination of RIP1 and polyubiquitin binding by NEMO. Molecular cell.

[12] Li H, Kobayashi M, Blonska M, You Y, Lin X (2006). Ubiquitination of RIP is required for tumor necrosis factor alpha-induced NF-kappaB activation. The Journal of biological chemistry.

[13] Vandenabeele P, Declercq W, Van Herreweghe F, Ven Berghe T (2010). The role of the kinases RIP1 and RIP in TNF-induced necrosis. Science signaling.

[14] Chen ZJ, Parent L, Maniatis T (1996). Site-specific phosphorylation of IkappaBalpha by a novel ubiquitination-dependent protein kinase activity. Cell.

[15] Ghosh S, Baltimore D (1990). Activation *in vitro* of NF-kappa B by phosphorylation of its inhibitor I kappa B. Nature.

[16] Kovalenko A, Chable-Bessia C, Cantarella G, Israel A, Wallach D, Courtois G (2003). The tumour suppressor CYLD negatively regulates NF-kappaB signalling by deubiquitination. Nature.

[17] Trompouki E, Hatzivassiliou E, Tsichritzis T, Farmer H, Ashworth A, Mosialos G (2003). CYLD is a deubiquitinating enzyme that negatively regulates NF-kappaB activation by TNFR family members. Nature.

[18] Sun SC (2010). CYLD: a tumor suppressor deubiquitinase regulating NF-kappaB activation and diverse biological processes. Cell death and differentiation.

[19] Wertz IE, O'Rourke KM, Zhou H, Eby M, Aravind L, Seshagiri S, Wu P, Wiesmann C, Baker R, Boone DL, Ma A, Koonin EV, Dixit VM (2004). De-ubiquitination and ubiquitin ligase domains of A20 downregulate NF-kappaB signalling. Nature.

[20] Evans PC, Ovaa H, Hamon M, Kilshaw PJ, Hamm S, Bauer S, Ploegh HL, Smith TS (2004). Zinc-finger protein A20, a regulator of inflammation and cell survival, has de-ubiquitinating activity. The Biochemical journal.

[21] Shembade N, Harhaj NS, Liebl DJ, Harhaj EW (2007). Essential role for TAX1BP1 in the termination of TNF-alpha-, IL-1- and LPS-mediated NF-kappaB and JNK signaling. The EMBO journal.

[22] Enesa K, Zakkar M, Chaudhury H, Luong le A, Rawlinson L, Mason JC, Haskard DO, Dean JL, Evans PC (2008). NF-kappaB suppression by the deubiquitinating enzyme Cezanne: a novel negative feedback loop in pro-inflammatory signaling. The Journal of biological chemistry.

[23] Song L, Lin C, Gong H, Wang C, Liu L, Wu J, Tao S, Hu B, Cheng SY, Li M, Li J (2013). miR-486 sustains NF-kappaB activity by disrupting multiple NF-kappaB-negative feedback loops. Cell research.

[24] L'Esperance S, Popa I, Bachvarova M, Plante M, Patten N, Wu L, Tetu B, Bachvarov D (2006). Gene expression profiling of paired ovarian tumors obtained prior to and following adjuvant chemotherapy: molecular signatures of chemoresistant tumors. International journal of oncology.

[25] Nikolaou K, Tsagaratou A, Eftychi C, Kollias G, Mosialos G, Talianidis I (2012). Inactivation of the deubiquitinase CYLD in hepatocytes causes apoptosis, inflammation, fibrosis, and cancer. Cancer cell.

[26] Ambros V (2004). The functions of animal microRNAs. Nature.

[27] Bartel DP (2004). MicroRNAs: genomics, biogenesis, mechanism, and function. Cell.

[28] Iha H, Peloponese JM, Verstrepen L, Zapart G, Ikeda F, Smith CD, Starost MF, Yedavalli V, Heyninck K, Dikic I, Beyaert R, Jeang KT (2008). Inflammatory cardiac valvulitis in TAX1BP1-deficient mice through selective NF-kappaB activation. The EMBO journal.

[29] Chen LF, Greene WC (2004). Shaping the nuclear action of NF-kappaB. Nature reviews Molecular cell biology.

[30] Naugler WE, Karin M (2008). NF-kappaB and cancer-identifying targets and mechanisms. Current opinion in genetics & development.

[31] Espinosa L, Cathelin S, D'Altri T, Trimarchi T, Statnikov A, Guiu J, Rodilla V, Ingles-Esteve J, Nomdedeu J, Bellosillo B, Besses C, Abdel-Wahab O, Kucine N, Sun SC, Song G, Mullighan CC (2010). The Notch/Hes1 pathway sustains NF-kappaB activation through CYLD repression in T cell leukemia. Cancer cell.

[32] Massoumi R, Kuphal S, Hellerbrand C, Haas B, Wild P, Spruss T, Pfeifer A, Fassler R, Bosserhoff AK (2009). Down-regulation of CYLD expression by Snail promotes tumor progression in malignant melanoma. The Journal of experimental medicine.

[33] Hellerbrand C, Bumes E, Bataille F, Dietmaier W, Massoumi R, Bosserhoff AK (2007). Reduced expression of CYLD in human colon and hepatocellular carcinomas. Carcinogenesis.

[34] Bignell GR, Warren W, Seal S, Takahashi M, Rapley E, Barfoot R, Green H, Brown C, Biggs PJ, Lakhani SR, Jones C, Hansen J, Blair E, Hofmann B, Siebert R, Turner G (2000). Identification of the familial cylindromatosis tumour-suppressor gene. Nature genetics.

[35] Orditura M, Galizia G, Sforza V, Gambardella V, Fabozzi A, Laterza MM, Andreozzi F, Ventriglia J, Savastano B, Mabilia A, Lieto E, Ciardiello F, De Vita F (2014). Treatment of gastric cancer. World journal of gastroenterology : WJG.

[36] Gilbert J, Lee JW, Argiris A, Haigentz M, Feldman LE, Jang M, Arun P, Van Waes C, Forastiere AA (2013). Phase II 2-arm trial of the proteasome inhibitor, PS-341 (bortezomib) in combination with irinotecan or PS-341 alone followed by the addition of irinotecan at time of progression in patients with locally recurrent or metastatic squamous cell carcinoma of the head and neck (E1304): a trial of the Eastern Cooperative Oncology Group. Head & neck.

[37] Andreopoulou E, Vigoda IS, Valero V, Hershman DL, Raptis G, Vahdat LT, Han HS, Wright JJ, Pellegrino CM, Cristofanilli M, Alvarez RH, Fehn K, Fineberg S, Sparano JA (2013). Phase I-II study of the farnesyl transferase inhibitor tipifarnib plus sequential weekly paclitaxel and doxorubicin-cyclophosphamide in HER2/neu-negative inflammatory carcinoma and non-inflammatory estrogen receptor-positive breast carcinoma. Breast cancer research and treatment.

[38] Orlowski RZ AS (2002). NF-kappaB as a therapeutic target in cancer. Trends in molecular medicine.

