# Supplementary material for: MicroRNA-500 sustains nuclear factor-κB activation and induces gastric cancer cell proliferation and resistance to apoptosis
Source: Oncotarget. 2015 Jan 30;6(4):2483–95. doi: 10.18632/oncotarget.2800 (PMC4385865; doi:10.18632/oncotarget.2800)
Supplement: Supplementary file 1 [file oncotarget-06-2483-s001.pdf]

SUPPLEMENTARY MATERIALS AND METHODS

Primers and siRNAs

BCL2L1-up: 5′-TTCAGTGACCTGACATCCCA-3′; BCL2L1-dn: 5′-CTGCTGCATTGTTCCCATAG-3′; CCND1-up: 5′-TCCTCTCCAAAATGCCAGAG-3′ CCND1-dn: 5′-GGCGGATTGGAAATGAACTT-3′; XIAP-up: 5′-GACCCTCCCCTTGGACC-3′; XIAP-dn: 5′-CTGTTAAAAGTCATCTTCTCTTGAAA-3′; GAPDH-up: 5′-ATTCCACCCATGGCAAATTC-3′; GAPDH-dn: 5′-TGGGATTTCCATTGATGACAAG-3′.

CYLD siRNA: 5′- CGCUGUAACUCUUUAGC AU-3′; TAX1BP1 siRNA: 5′-GCACAA CAUGAAAGA GAACAA-3′; OTUD7B siRNA: 5′-CCUGUAUAUGA GAGCCUUGAA-3′.

SUPPLEMENTARY FIGURES AND TABLE

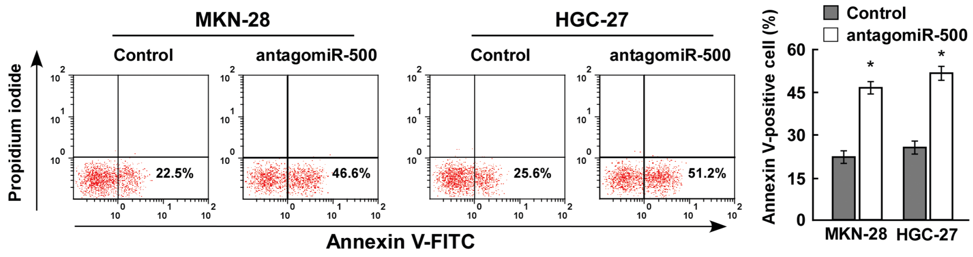

Supplementary Figure 1: Annexin V–FITC/PI staining of cells treated with cisplatin (20 µM) for 12 h.

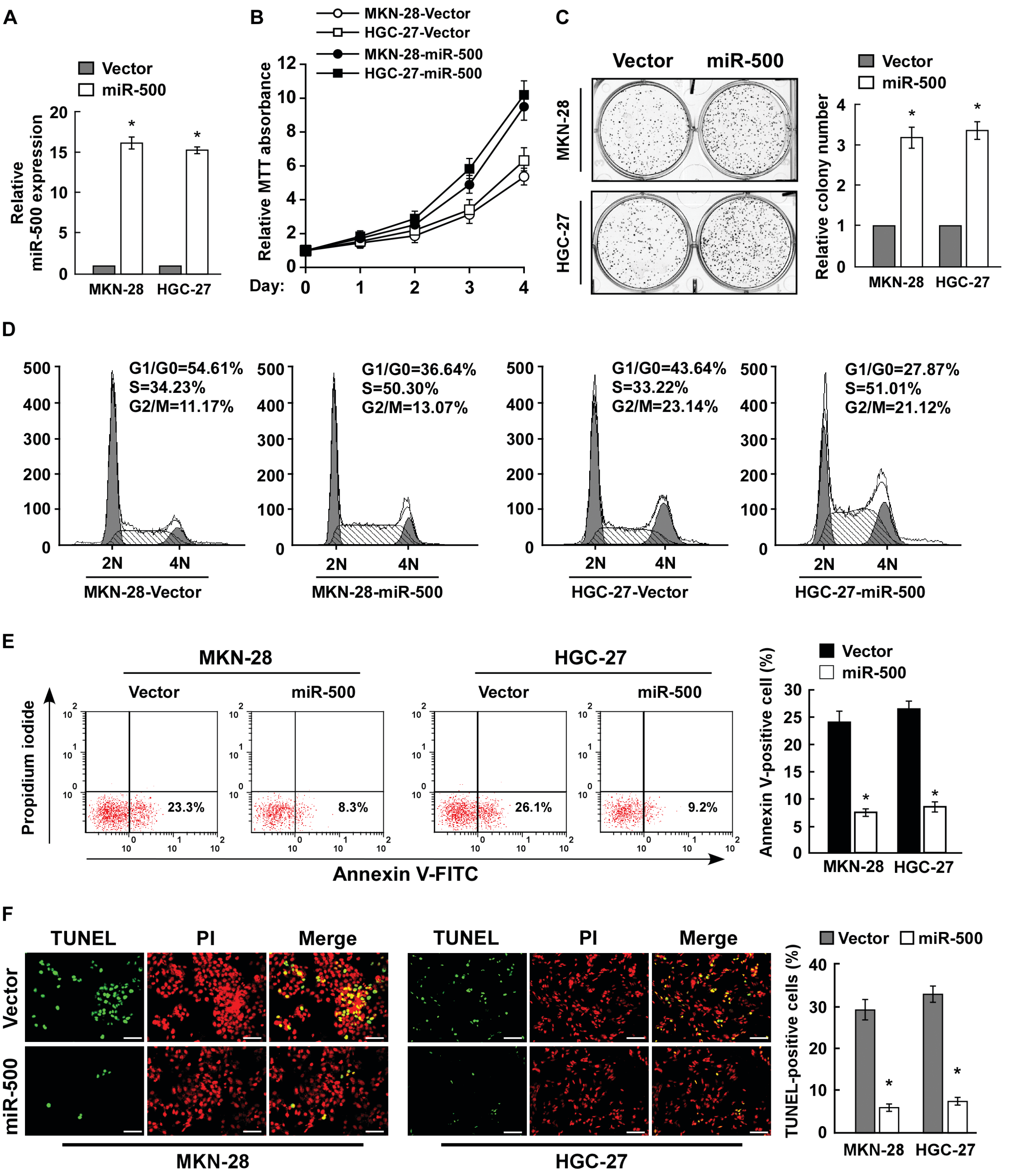

Supplementary Figure 2: Overexpression of miR-500 promotes proliferation and reduces apoptosis of gastric cancer cells *in vitro*. (A) Real-time PCR analysis of miR-500 expression in miR-500–overexpressing cells. Transcript levels were normalised by *U6* expression. **(B)** MTT assay revealing that miR-500 upregulation induced proliferation of MKN-28 and HGC-27 cells. **(C)** Representative micrographs (left) and quantification (right) of crystal violet–stained cell colonies. **(D)** Flow cytometry cell cycle analysis of gastric cancer cells. **(E)** Annexin V–FITC/PI staining of cells treated with cisplatin (20 μM) for 12 h. **(F)** Representative micrographs (left) and quantification of TUNEL-positive cells in cells treated with cisplatin (20 μM) for 36 h. Scale bars: 50 μm. Each bar represents the mean of three independent experiments. $^{*}p < 0.05$.

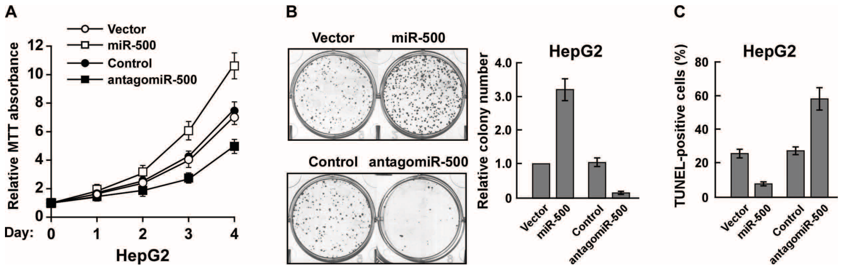

Supplementary Figure 3: miR-500 promotes cell proliferation and survival in HepG2 cell. **(A)** MTT assay revealing that miR-500 overexpression promoted, but miR-500 downregulation inhibited proliferation of HepG2 cell. **(B)** Representative micrographs (left) and quantification (right) of crystal violet–stained colonies. **(C)** TUNEL-positive cells in indicated cells treated with cisplatin (20 µM) for 36 h. Each bar represents the mean of three independent experiments.

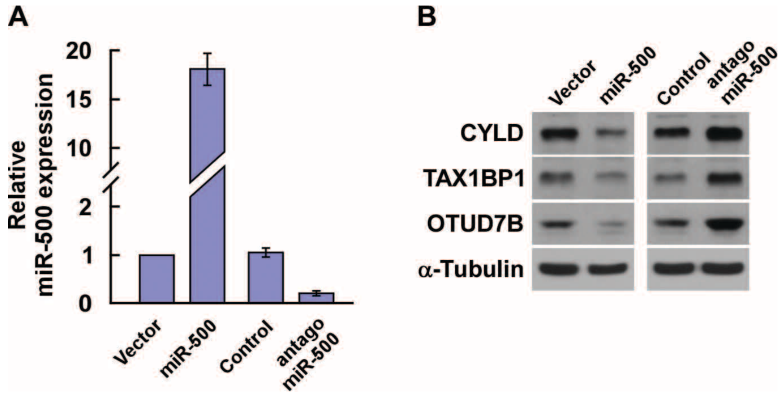

Supplementary Figure 4: Overexpression of miR-500 contributes to gastric cancer progression *in vivo*. **(A)** Real-time PCR analysis of miR-500 expression in indicated tumors. Transcript levels were normalised by *U6* expression. **(B)** Western blots of CYLD, TAX1BP1, and OTUD7B expression. α-Tubulin served as the loading control.

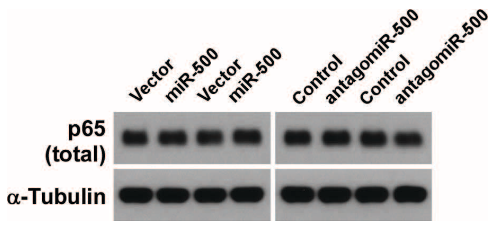

Supplementary Figure 5: Total p65 expression in miR-500-overexpressing and –silenced cells compared to controls. α-Tubulin served as the loading control.

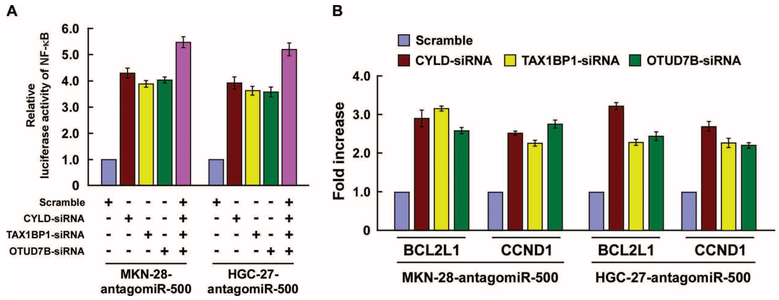

Supplementary Figure 6: Silencing CYLD, TAX1BP1, or OTUD7B in miR-500–inhibited cells recovered NF-κB activity (A), BCL2L1 and CCND1 expression (B). Each bar represents the mean ± SD of three independent experiments. $*p < 0.05$.

Supplementary Table 1: Clinicopathological characteristics of studied patients and expression of miR-500 in gastric cancer

Factor	NO.	(%)
**Gender**		
Male	105	73.9
Female	37	26.1
**Age (years)**		
≤60	69	48.6
>60	73	51.4
**Clinical stage**		
I	6	4.2
II	31	21.8
III	84	59.2
IV	21	14.8
**T classification**		
T_1	7	4.9
T_2	37	26.1
T_3	26	18.3
T_4	72	50.7
**N classification**		
N_O	34	23.9
Yes	108	76.1
**M classification**		
No	121	85.2
Yes	21	14.8
**Histological differentiation**		
Well	131	92.3
Poor	11	7.7
**Vital status**		
Alive	45	31.7
Dead	97	68.3
**Expression of miR-500**		
Low expression	71	50.0
High expression	71	50.0

Supplementary Table 2: Correlation between the clinicopathological features and expression of miR-500

Patient characteristics		miR-500 expression		*p*-value
		Low	High	
**Gender**	Male	49	56	0.181
	Female	22	15	
**Age (years)**	≤60	36	33	0.614
	>60	35	38	
**Clinical stage**	I	6	0	< 0.001
	II	22	9	
	III	38	45	
	IV	4	17	
**T classification**	T_1	6	1	< 0.001
	T_2	29	8	
	T_3	16	10	
	T_4	20	52	
**N classification**	No	23	11	0.018
	Yes	48	60	
**M classification**	No	69	52	< 0.001
	Yes	2	19	
**Histological differentiation**	Well	69	62	0.028
	Poor	2	9	
**Vital status**	Alive	39	15	< 0.001
	Dead	41	56	

Supplementary Table 3: Univariate and multivariate analysis of different prognostic parameters in patients with gastric cancer by Cox-regression analysis

	Univariate analysis		Multivariate analysis	
	p	Hazard ratio (95% CI)	*p*	Hazard ratio (95% CI)
**Clinical stage**	< 0.001	1.621 (1.353–1.921)	0.021	0.932 (0.635–1.234)
I				
II				
III				
IV				
**T classification**	< 0.001	1.521 (1.192–1.832)	0.031	1.621 (1.170–1.933)
T_1				
T_2				
T_3				
T_4				
**N classification**	0.031	2.013 (1.625–2.756)	0.056	1.936 (1.035–2.872)
No				
Yes				
**M classification**	< 0.001	2.638 (1.815–3.357)	0.004	2.032 (1.456–3.031)
No				
Yes				
**miR-500 expression**	< 0.001	1.927 (1.435–2.820)	< 0.001	2.234 (1.662–3.232)
Low expression				
High expression				
